# Frailty and stroke: Global implications for assessment, research, and clinical care—A WSO scientific statement

**DOI:** 10.1177/17474930251345295

**Published:** 2025-05-20

**Authors:** Nicholas R Evans, João Pinho, Lucy Beishon, Tu Nguyen, Aravind Ganesh, Bharathi Balasundaram, Ragnhild Munthe-Kaas, Jonathan Hewitt, Dorcas B C Gandhi, Terry J Quinn, Richard I Lindley

**Affiliations:** 1 Department of Clinical Neurosciences, University of Cambridge, Cambridge, UK; 2 RWTH Aachen University, Braga, Germany; 3 University of Leicester, Leicester, UK; 4 The George Institute for Global Health, University of New South Wales, Sydney, NSW, Australia; 5 Departments of Clinical Neurosciences and Community Health Sciences, The Hotchkiss Brain Institute, the Mathison Centre for Mental Health Research and Education, Cumming School of Medicine, University of Calgary, Calgary, AB, Canada; 6 The O’Brien Institute for Public Health, Cumming School of Medicine, University of Calgary, Calgary, AB, Canada; 7 Department of Psychological Medicine, Changi General Hospital, Singapore; 8 Department of Medicine, Kongsberg Hospital, Vestre Viken Hospital Trust, Oslo, Norway; 9 Cardiff University, Cardiff, UK; 10 Christian Medical College & Hospital, Ludhiana, PB, India; 11 Manipal Academy of Higher Education, Manipal, India; 12 School of Cardiovascular and Metabolic Health, University of Glasgow, Glasgow, UK; 13 The University of Sydney, Australia

**Keywords:** Acute stroke therapy, ischemic stroke, hemorrhage, rehabilitation, reperfusion, risk factors, stroke, stroke prevalence, treatment, frailty

## Abstract

Frailty is common in stroke and has important disease- and treatment-modifying effects. The need to develop clinical practice and research for the impact of frailty on stroke is likely to increase in the coming decades as the global population ages, resulting in a higher burden of frailty that is likely to be borne disproportionately by lower- and middle-income countries.

The global nature of frailty in stroke necessitates global action. This World Stroke Organization Scientific Statement synthesizes the current evidence relating to the prevalence and effects of frailty across the stroke pathway. Furthermore, it includes expert consensus on priority areas from a global panel: standardization of frailty assessments for research, explicit measurements of frailty (in addition to disability) in large clinical trials, dedicated studies investigating the treatment-modifying effects of frailty in acute stroke and secondary prevention, research investigating the impact of frailty on the different aspects of recovery and rehabilitation after stroke, and understanding the mechanisms underpinning the relationship between frailty and stroke for potential therapeutic exploitation.

This scientific statement has been reviewed and approved by the World Stroke Organization Executive.

## Graphical abstract

**Graphical abstract:**
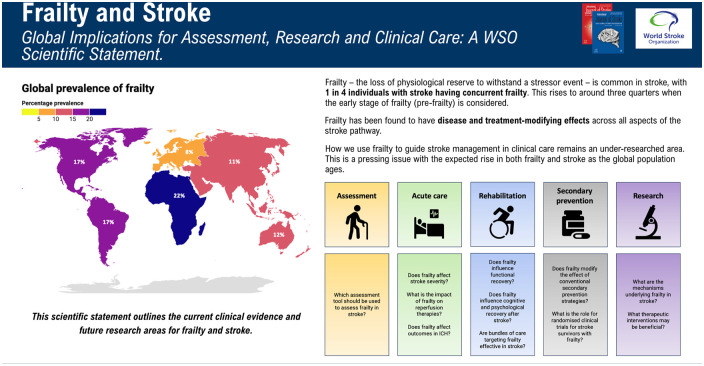

## Introduction

Between 2020 and 2050, the global trend in the aging population will see a doubling of those aged over 60 years (reaching 2.1 billion) and a three-fold increase in those aged over 80 years (reaching 426 million). Although initially driven by increases in high-income countries, by 2050 it is predicted that two-thirds of the global population aged over 60 years will be found in low- and middle-income countries.^1^

The aging population will see a rise in the prevalence of frailty. Frailty is characterized by the loss of physiological reserve to withstand a stressor event, resulting in increased susceptibility to illness. Furthermore, across most acute medical presentations, the illness that the individual with frailty experiences is typically more severe, longer, and with less complete recovery than in more robust individuals.^2^ Stroke is no exception, with frailty found to have both disease- and treatment-modifying effects.

Frailty is a clinical syndrome that, although closely related to the aging process, is distinct from chronological age. Importantly, frailty is also distinct from disability and multimorbidity, though considerable overlap exists between the three. Despite previous optimism that we would see “compression of morbidity” with improving healthcare, recent evidence indicates that this is not occurring in many countries, suggesting that multimorbidity is here to stay.^3^ Whether a similar “compression of frailty” exists for the time lived with frailty, and whether this may provide a more effective target for promoting healthy aging, has important implications for global health.

**Figure 1.**
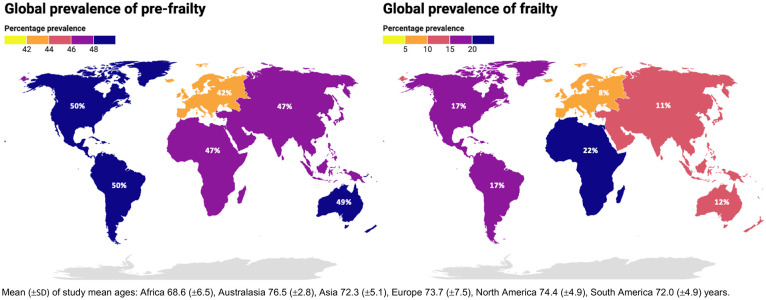
Global prevalence of pre-frailty (left) and frailty (right), and mean age of included study mean ages. Data from O’Caoimh et al.^4^

The global prevalence of frailty is 12–24% among those aged over 50 years of age, while the prevalence of pre-frailty (a prodromal intermediate state before frailty becomes established, and which is potentially reversible) is higher at 46–49%.^4^ In the same meta-analysis, the prevalence of both pre-frailty and frailty rose with age: frailty rose from 11% for individuals 50–59% to 51% for those aged 90+ years of age.^4^ Furthermore, frailty is more prevalent in lower- and middle-income countries versus high-income countries among those aged over 50 years of age (Figure 1).^4^ However, accurate comparisons are challenging given most frailty research occurs in high-income countries (36.2% of datasets come from Europe, compared with 2.6% from Africa), with the paucity of research in lower-/middle-income countries only recently starting to be addressed.^5^

A recent meta-analysis demonstrated a pooled prevalence of frailty of 24.6% among individuals presenting with stroke, with broadly comparable prevalence across the United Kingdom, Europe, North America, and Australasia.^6^ The pooled prevalence of frailty in stroke is 15% in Asia,^7^ with no data available from Africa. The prevalence of frailty has also risen among stroke survivors: in the United States, England, and Europe, the prevalence of frailty in those with a previous stroke was 23.8–54.6% depending on the assessment method.^8^

**Figure 2.**
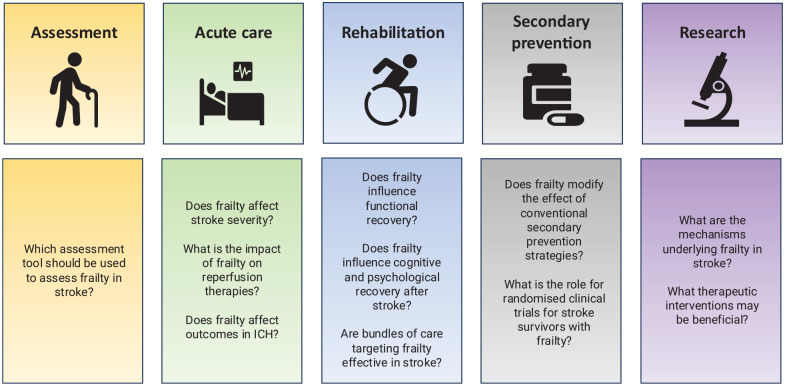
Key research and clinical priorities.

The aging population and predicted increase in stroke incidence represents a significant global public health challenge. This World Stroke Organization Scientific Statement on Frailty in Stroke sought expert consensus on the current state of the field, including key areas of uncertainty for future research and clinical priorities in acute, rehabilitation, and secondary prevention settings (Figure 2).

## Assessment of frailty in stroke

There are two broad approaches to measuring frailty: a phenotype-based approach or the cumulative deficit model. The former—comprising weight-loss, self-reported exhaustion, low levels of physical activity, slow gait speed, and weak grip strength—has demonstrated a modifying effect on disease trajectories across a range of medical conditions.^9^ However, these features can be challenging to assess in the acute stroke setting, and also do not consider cognition and psychosocial aspects explicitly.

In contrast, the cumulative deficit model reflects the approach that the accumulation of pathology over the life-course results in multisystem physiological impairment and frailty. This may be measured using a frailty index (FI); a list of possible salient deficits encompassing physiological and functional parameters, as well as comorbidities.^10^ An electronic frailty index (eFI) has also been developed for use in electronic health record data. The methodology to derive an FI has been well-described.^11,12^ A FI relating to stroke has been formulated, validated, and used in a number of stroke studies to date.^6,13,14^

The principle of cumulative deficit is also reflected in the Hospital Frailty Risk Score (HFRS). This method for assessing frailty in an unselected cohort uses 109 diagnostic codes from the International Classification of Disease relating to frailty. Higher scores are associated with an increased risk of stroke,^15^ as well as poorer outcomes.^16–18^ However, the HFRS has only been validated using Hospital Episode Statistics data, and its applicability in other healthcare datasets/systems requires further investigation.

Another commonly used assessment tool in clinical practice is the Clinical Frailty Scale (CFS). This ordinal scale is validated for use in individuals aged over 65 years of age, and involves an in-person clinical assessment of an individual’s function, physical activity, energy, and mobility in the 2 weeks prior to an acute illness.^19^

### Relationship to modified Rankin scale

Particular mention should be made of a common misconception relating to the use of the modified Rankin scale (mRS) to measure frailty. mRS is a disability scale and has only a moderate correlation with FI, indicating disability and frailty are distinct.^14,20,21^ This is further exemplified by studies reporting frailty measures were superior predictors of outcomes than mRS.^14,22,23^

### Radiological assessment of frailty

There is also growing interest in neuroimaging markers of frailty (termed “brain frailty”), consisting of the chronic burden of vascular and neurodegenerative changes present in the brain.^24,25^ Many brain frailty markers can be identified on routinely acquired neuroimaging: cortical or subcortical atrophy, white matter hyperintensities or leukoaraiosis, lacunes, prior infarcts, microbleeds, cortical superficial siderosis, and enlarged perivascular spaces.^24,25^ These markers may affect the ability of the brain to compensate for acute vascular injuries, conferring additional vulnerability to functional and cognitive decline, analogous to the cumulative deficit model of clinical frailty.^10^

Brain frailty in individuals with stroke is very high, with one meta-analysis reporting the prevalence as high as 73.8%.^26^ However, correlations between brain frailty and either CFS or frailty phenotype for individuals with transient ischemic attack (TIA) and ischemic stroke were weak (0.336 and 0.23 respectively), indicating that brain frailty and physical frailty are distinct entities, albeit ones that frequently coexist.^27^

### Future directions

The most appropriate measure of frailty is likely to vary according to the setting.^28^ For a frailty assessment tool to be effective in the time-pressured clinical setting, it will need to be time-efficient and possible to use with the amount of information typically available in acute presentations. The CFS is likely to provide the most suitable means for this, as it may be scored quickly using routinely collected clinical information. It may also be more feasible in settings with lower healthcare resources, particularly those lacking electronic record systems. Training is available, and the CFS is available in a number of languages and pictorial forms.

In less time-pressured settings and research studies, an index-based approach may provide a greater spread of values allowing greater sensitivity in statistical analysis. The use of diagnostic codes in the HFRS may also be more conducive to retrospective frailty scoring, and indeed the nature of the FI and HFRS makes it more likely that these will be calculated retrospectively rather than informing care in real time. However, currently funded research is likely to lead to automated frailty scales, using data from government, healthcare organizations, and electronic medical records, with the vision of a pop-up summary of frailty when opening a patient’s medical record.

Radiological assessment of brain frailty holds promise for automation and integration into clinical workflow, as well as the potential to be combined with clinical assessments of frailty as part of an enhanced radiomics approach for clinical application.

With this in mind, we suggest the use of the following assessment tools in the following contexts (Table 1):

**Table 1.** Frailty assessment tools according to context.

Research use	FI/eFI/HFRS for establishing relationships. CFS for translational research.
Clinical use	CFS in most settings. FI and HFRS may be feasible when electronic records are available.
For use with population records	eFI, HFRS.
Radiological assessment	Radiological markers of ‘brain frailty.’

**Figure 3.**
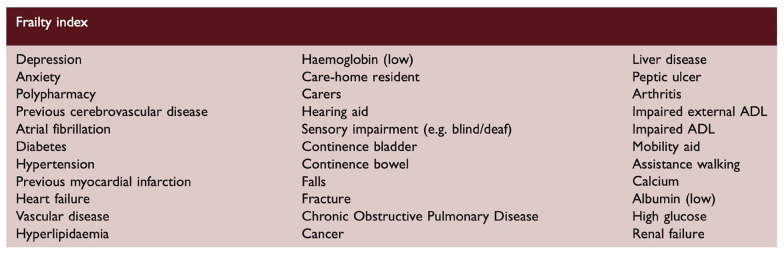
FI proposed by Taylor-Rowan et al.^21^ Reproduced with permission from Evans et al.^29^

As will be seen in subsequent sections, the wide variety of assessment scales used to measure frailty—as well as variation in their operationalization in practice (including considerations such as variability in hospital administrative coding underpinning the HFRS and errors in the timing of assessment relating to baseline frailty—poses a challenge for comparing studies and generating meta-analyses. This is further compounded by the inclusion of different deficits in the FIs used in different studies. Standardization of approaches and components of FIs would be highly beneficial. In this regard, the FI proposed by Taylor-Rowan et al.^21^ has been validated in a stroke population and used in subsequent studies, and considers the range of domains required for a robust FI (Figure 3).

## Pre-stroke frailty and acute stroke

### Frailty and risk of stroke

According to a large meta-analysis, the pooled relative risk of stroke for individuals with frailty compared with those without frailty is 1.72.^30^ Mendelian randomization studies found that genetically predicted frailty was associated with ischemic stroke, in particular large artery atherosclerosis etiology.^31^ This may reflect the rise in the prevalence of cardiovascular risk factors with frailty.^17^ In individuals with atrial fibrillation (AF), concurrent frailty—but not pre-frailty—approximately tripled the risk of stroke.^32^

### Frailty and stroke severity

The association between pre-stroke frailty and initial stroke severity remains unclear. Some studies have reported a non-significant relationship with stroke severity (National Institutes of Health Stroke Scale, NIHSS) on multivariable analysis, though there was a trend toward significance.^21,33^ In contrast, other studies report an association that remained significant on multivariable analysis.^34^ In a cohort of lacunar strokes, those with severe frailty were approximately 2.5-times more likely to have a severe stroke than individuals with non-severe frailty.^35^ Other studies in ischemic strokes of embolic etiology have also reported a significant but weak association between FI and NIHSS.^14^

These disparate findings may reflect the different methods of assessing frailty, limited sample size, and heterogeneous controlling for bias. Furthermore, the effects of cognitive impairment and/or radiological assessments of the aforementioned “frail brain” in mediating the relationship between frailty and stroke severity have typically not been considered. Further work in a larger cohort would be advantageous for establishing the relationship, the mechanistic underpinnings of which may represent therapeutic targets in either the pre-stroke or acute settings.

### Early mortality after ischemic stroke

A number of studies have shown that pre-stroke frailty is associated with mortality at 1–3 months following ischemic stroke.^18,33,36^ Across approximately two million stroke admissions (both ischemic and hemorrhagic strokes), increasing frailty was independently associated with a higher likelihood of in-hospital mortality (adjusted odds ratios (aORs) of 1.62 for pre-frail and 2.82 for frail).^17^ A similar result was seen in a cohort of lacunar strokes.^35^

### Complications after stroke

Pre-stroke frailty is associated with acute kidney injury,^17^ urinary tract infection,^35^ post-stroke pneumonia,^35^ and delirium.^21^ The odds of recurrent stroke within 2 years were increased by 50–70% for individuals with concurrent stroke and frailty, but not for those with pre-frailty.^8^ The length of hospital admission after stroke is also longer in individuals with premorbid frailty.^35^

### Frailty and reperfusion therapies

Individuals with frailty are less likely to receive thrombolysis.^35^ In a proof-of-principle study, individuals with frailty had a reduced response to thrombolysis, with each additional point on the CFS attenuating the benefit from thrombolysis by one NIHSS point.^33^

Similar attenuated responses for individuals with frailty have been described in mechanical thrombectomy, though most data come from single-center observational studies using dichotomized measures of frailty. In a UK study, those with pre-stroke frailty were 3 times more likely to die or be left dependent after thrombectomy than those without pre-stroke frailty.^37^ In a Finnish study, frail individuals aged over 80 years had similar rates of recanalization and bleeding complications to those without frailty, but had higher rates of mRS 4–6.^38^ Similar trends of poorer 24-h NIHSS, increased in-hospital mortality, and 1-year mortality according to pre-stroke frailty were seen in studies from Germany.^39,40^

In a single-center German study, individuals with a mRS 0–2 three months after thrombectomy had lower levels of HFRS at hospital discharge, and a high frailty risk was independently associated with a halving in the odds of a favorable functional outcome.^16^

In a mediation analysis, frailty was estimated to account for 28% of the total effect of age on poor functional outcomes (mRS 3–6) and death following reperfusion therapies for acute ischemic stroke.^41^ In another mediation analysis of the ESCAPE-NA1 clinical trial, neuroimaging markers of frailty were estimated to account for 85.1% for the total effect of age on functional outcomes after thrombectomy.^42^ Potential mechanisms are discussed in later sections.

Although individuals with frailty are less likely to receive reperfusion therapies, the fundamental question remains of what we should be doing for such individuals. An attenuated response may not mean that there is no clinically significant benefit for the individual (for example, allowing an individual to return home). Rather than assume treatment nihilism, we need higher-quality data to guide the appropriate use of reperfusion therapies in this population.

### Hemorrhagic stroke

In contrast to ischemic stroke, there is a paucity of data on the relationship between pre-stroke frailty and outcomes after intracerebral hemorrhage (ICH), and data have produced conflicting results.

A single-center study in the United States found no difference in in-hospital mortality after ICH between those with and without pre-stroke frailty (though the results approached statistical significance and may reflect the sample size and assessment method used).^43^ In contrast, data from a UK center found an incremental increase in the FI of 0.1 was independently associated with an aOR of 1.09 for 28-day mortality.^13^ In a larger Singaporean study, the frailty-associated impact on 30-day mortality was present for men but not women.^44^ Premorbid frailty is also associated with a poorer recovery (mRS 3–6) after ICH, with a stronger association for women than men (odds ratios (ORs) of 2.19 and 1.81).^44^

The small number of studies means that a large observational study would be advantageous in establishing the definitive relationship between pre-stroke frailty and outcomes after ICH, and whether this is affected by ICH location/etiology. Given the disproportionately high burden of disability from ICH and the predicted rise in its incidence with an aging population, understanding the effects of frailty on ICH outcomes warrants urgent attention.

### Frailty in TIA

Frailty is common among a cohort attending a single UK center for assessment for a TIA, with the finding that 53.5% of individuals were frail at baseline, and that this prevalence rises after TIA.^45^ However, despite the prevalence of frailty among individuals with TIA, there has been little research done on its relationship to date. The impact of systemic frailty and/or the “frail brain” on TIA severity and long-term sequelae is an important area for future research.

### Future directions

Multiple studies have reported the association between frailty and poorer outcomes across stroke subtypes and treatments. However, many are relatively small single-center studies that consider dichotomized/tertiled frailty and dichotomized outcomes. Frailty is a spectrum, and it is arguably reductionist to consider it as a binary condition, potentially losing the information relating to the most challenging cases of intermediate frailty. Future work would benefit from larger multicentre prospective studies using a continuous scale (see above).

## Frailty in stroke rehabilitation and recovery

Disease- and treatment-modifying effects of frailty in rehabilitation may reflect the attenuated neurological recovery in the acute setting, but may also reflect the multisystem effects of frailty affecting rehabilitation directly, as well the majority of frail stroke survivors not being prioritized for rehabilitation.^17^

### Functional recovery

A recent meta-analysis investigating frailty and functional outcomes highlighted the global interest in this topic, with studies from Europe (12 studies), Asia (8 studies), North America (3 studies), and Australia (2 studies).^7^ This revealed that frailty was associated with a 1.21-higher odds of ending up with a poor functional outcome.^7^ This effect was consistent across geographic regions, highlighting the global impact of frailty on stroke recovery.

In the multicenter Nor-COAST study, both the pre-stroke FI and mRS were associated with unfavorable mRS 3–6 at 3 years, with the pre-stroke FI being a stronger predictor (ORs 4.1 and 2.7 respectively).^23^ Similar results for pre-frailty and frailty have been reported in Chinese and Japanese populations.^46–48^

Radiological measures of brain frailty have been independently associated with unfavorable shifts in mRS at 90 days for all strokes.^49^ The IST-3 Group have also looked at the effect of brain frailty, finding that features of brain frailty had important predictive value.^50^ These results were confirmed in the ENCHANTED dataset, despite being a younger ischemic stroke cohort.^51^

### Cognition after stroke

Pre-stroke frailty was independently associated with post-stroke cognitive impairment in one UK study, and was further exacerbated by age, delirium, pre-stroke cognitive impairment, and stroke severity.^52^ Similar findings were reported by Munthe-Kaas et al,^22^ with frailty proving a stronger predictor than pre-stroke mRS for post-stroke neurocognitive disorder at 3 months after stroke (OR = 3.09 and 2.21 respectively). However, in a study from China, although the relationship between physical frailty and poorer post-stroke cognition remained significant after adjustment for cardiovascular risk factors, the relationship became non-significant when baseline cognitive scores were included, indicating the importance of considering this pre-stroke baseline.^53^

The presence of radiologically assessed brain frailty was associated with worse cognitive scores at 90 days for all strokes and for those with lacunar strokes, with the relationship being stronger for those with lacunar strokes.^49^ Brain frailty on baseline imaging, CFS, and Fried frailty phenotype were each independently associated with cognitive impairment at 18 months following the stroke event.^27^

In a sample of individuals with asymptomatic chronic small vessel disease, those subsequently developing dementia were more likely to be pre-frail/frail, but exclusively for a mobility frailty phenotype.^54^ This may represent the vicious cycle between small vessel disease, gait impairment, frailty, and cognition. Others have found an important interaction of frailty with other forms of neurological damage suggesting that frailty might contribute to cognitive impairment in other more fundamental ways.^55^

### Psychological outcomes

There has been relatively little research into the impact of frailty on psychological outcomes in the post-stroke setting. One study found Hospital Anxiety and Depression Scale scores were significantly higher at 6 months after stroke for individuals with pre-stroke frailty compared with non-frail individuals.^56^ This is compounded by a reduced response to psychosocial treatment.^57^

Reflecting this, frailty appears to be associated with worse health-related quality of life after stroke,^56,58^ which is associated with pronounced decline with direct effects on rehabilitation outcomes.

### Feeding after stroke

Similarly, there has been little research on the impact of frailty on long-term feeding after stroke. A small pilot study showed an increased 1-year mortality after percutaneous endoscopic gastrostomy tube insertion for those with pre-stroke frailty.^59^

### Discharge destination

Studies from the Netherlands and China report no difference in post-stroke discharge destination between pre-stroke frail and non-frail cohorts.^56,60^ In contrast, a study of stroke survivors from a center in Tokyo found a significantly higher proportion returning home after stroke among non-frail versus frail individuals (81% vs. 12%).^48^ In a study from the United States, this proportion was lower in both non-severely frail (43.5%) and severely frail cohorts (35.5%).^35^

Compared with stroke survivors with frailty, individuals with pre-frailty and no frailty were 52% and 72% more likely to be discharged to an in-patient rehabilitation facility in a study of Medicare recipients in the United States.^61^

### Long-term mortality

Increasing frailty after stroke is associated with an increased risk of mortality at 1 year.^62,63^ Furthermore, pre-stroke frailty remains associated with mortality at 2 and 3 years post-stroke.^8,23^

### Future directions

Although there has been a marked increase in interest around the role of frailty in stroke rehabilitation and recovery, the complex nature of rehabilitation means that there remain many unknowns. To date, there have been no studies considering a disease-modifying effect of frailty relating to polypharmacy (typically defined as the use of five or more mediations concurrently), aphasia recovery, apathy, and fatigue. The final two considerations may be particularly pertinent, given exhaustion and low physical activity are defining frailty phenotypes.

Further avenues for investigation include the operationalization of frailty status for improving rehabilitation prognostication and goal-setting, and the development of frailty-specific rehabilitation programs. Ultimately, better prognostication for stroke survivors with frailty may help inform their preferences and enhance shared decision-making relating to rehabilitation goals, and is explored further below.

## Frailty and secondary prevention

Despite its importance, there has been relatively little research into whether frailty exerts a treatment-modifying effect on secondary prevention strategies after stroke. Recent work has demonstrated that it has been possible to estimate frailty retrospectively in some previous trials, and post hoc analyses have shown variable treatment-modifying effects from frailty.^64^ This variation in post hoc analyses highlights the importance of prospectively measuring frailty to confirm or refute such interactions. In order to highlight current areas of uncertainty, this Scientific Statement considered relevant outcomes from primary prevention studies, as well as studies considering secondary prevention in older age.

### Antiplatelet therapy

No studies have evaluated antiplatelet therapy after stroke according to frailty status (only by age). As risk-benefit ratios may alter frailty, studies considering initiation and duration of antiplatelet therapy for secondary prevention in individuals with frailty should be a focus of future research.

### Statins

In the primary care setting, statin use in individuals aged over 75 living with frailty was not associated with a significant reduction in all-cause mortality.^65^ No study has evaluated the impact of frailty on the efficacy of statins for reducing the risk of recurrent stroke.

### Hypertension

For individuals aged 75–84 years in primary care, systolic blood pressure readings above 140 mm Hg are associated with an increase in the risk of stroke for non-frail and mildly frail individuals, but not for those with moderate/severe frailty.^66^ In a meta-analysis of secondary prevention, intensive blood pressure control was associated with a reduced risk of recurrent stroke for those younger than 80 years of age, but not for older individuals.^67^ However, to date the effect of frailty in a cohort of stroke survivors has not been considered.

### Diabetes

Metabolic syndrome itself has been found not to relate to frailty, though the inflammatory component of metabolic syndrome was.^68^ To date, there has been no work considering the relationships between frailty, metabolic syndrome, and inflammation in relation to the impact on stroke recurrence and secondary prevention.

### Revascularization surgery

In a large registry of individuals undergoing carotid endarterectomy or stenting, 4.2% were frail. Frailty was independently associated with an increased risk of periprocedural stroke (aOR = 1.66) and death (aOR = 1.59).^69^

### Anticoagulation

In a large primary care dataset, the rate of composite death/stroke/systemic embolism/major bleeding rose with increasing frailty in those not receiving anticoagulation, and anticoagulation was associated with a reduction in the composite outcomes for each frailty category compared with those not on anticoagulation.^70^ Other studies have reported similar reductions in ischemic stroke without a significant increase in major bleeding complications in frail individuals, though the reduction in vascular events was no longer significant in the most severely frail individuals.^71,72^

Overall, the finding in a primary prevention setting that anticoagulation is associated with a reduction in vascular events without a significant increase in bleeding complications among frailer individuals would suggest that anticoagulation should not be withheld in individuals on the basis of frailty alone. However, validation in a cohort of stroke survivors with explicit consideration of frailty would be advantageous for confirming this.

### Future directions

Robust studies investigating any modulation from frailty on the efficacy of secondary prevention studies are urgently needed.^73^ Furthermore, many studies have considered stroke as a homogeneous entity, and consideration of the stroke etiology may reveal whether it modulates the impact of frailty on the efficacy of secondary prevention strategies. Finally, most of the aforementioned studies are observational in nature, and definitive randomized clinical trials in frail cohorts would be advantageous. These studies are crucial for ensuring that secondary prevention is targeted appropriately for stroke survivors with frailty; either by reducing polypharmacy where individuals are unlikely to benefit, or combating treatment nihilism by ensuring that individuals with frailty still receive medication from which they are likely to benefit.

## Future research

The importance of frailty has been recognized as a research priority by funders, national governments, and international organizations. In the United Kingdom, the Stroke Association-James Lind Alliance Priority Setting Partnership identified frailty as an area of unmet research need.^74^ As the global population ages, the need for such research is likely to increase.

A significant challenge for developing clinical care for individuals with stroke and concurrent frailty is the absence of robust evidence to shape treatments. Historically, large multicentre clinical trials have rarely measured frailty explicitly. Furthermore, many acute stroke studies excluded those with frailty by proxy, excluding such individuals on the basis of age or premorbid mRS. Consequently, there is a risk that the evidence being produced may not apply to a considerable proportion of those seen in clinical practice.

Given the increased recognition of the importance of frailty, frailty should be measured routinely in research practice (particularly in large pragmatic clinical trials). However, it is likely that evaluating the effects of frailty may be more complex than simply collecting frailty status as an additional variable for multivariable analysis, and that randomized clinical trials dedicated specifically to individuals affected by both frailty and stroke may be required, covering treatments across the whole stroke pathway.

In addition to the need for studies investigating associations between frailty and aspects of stroke treatment and rehabilitation, there are two further broad areas of study needing attention: research into the mechanisms by which frailty may influence stroke outcomes, and trials into innovative rehabilitation strategies to treat both stroke and frailty.

### Potential mechanisms for the effect of frailty on stroke

Relevant to the hyperacute setting, frailty is independently associated with reduced penumbral volumes, with a suggestion that this is driven by frailty-associated inflammation.^14^ Inflammation, particularly in relation to vascular disease and risk factors, may also “prime” the brain for injury.^75,76^ The hypothesized inflammatory basis to both vascular disease and frailty offers the possibility of targeting both using anti-inflammatory strategies.

Other mechanisms seen in frailty may also play a role in affecting stroke outcomes, including impaired cerebral hemodynamics and poorer leptomeningeal collateral flow.^77^ Further work in stroke populations would help elicit the relevance in symptomatic cerebrovascular disease, as well as whether it may represent a therapeutic target.

### Adapting stroke rehabilitation for individuals with frailty

The finding that some rehabilitation strategies benefit non-frail individuals, but not those with frailty, should not necessarily lead to the conclusion that rehabilitation is futile for stroke survivors with frailty, but rather rehabilitation programs may require adaptation to target frailty alongside the neurological deficit. Such approaches have been used in other conditions,^78^ and offer proof-of-principle for similar strategies in cerebrovascular disease. Interventions have included not only physical rehabilitation, incorporating targeted approaches to sarcopenia, but also nutritional and psychological interventions relevant to frailty. Future research must consider the feasibility and acceptability of such multidisciplinary interventions for stroke survivors with frailty prior to evaluation of their efficacy.

## Policy and practice implications of frailty in stroke care

The global nature of frailty necessitates global action. The heterogeneous character of frailty, incorporating not only physiological considerations but also psychosocial considerations, means that research and clinical practice must be representative of all settings in which stroke and frailty is found. The predominance of frailty research conducted in more economically developed countries, despite the predicted trend that most individuals with frailty will live in middle/low economically developed countries, means that the research may not be applicable to the majority of those who need it.^1,5^ We feel that this issue now requires international collaboration, dedicated studies with standardized assessments, with targeted representation and promotion of research and clinical practice in all settings where frailty and stroke occur. Ultimately, the over-riding challenge will be in implementing these findings at individual and population levels.

The anticipated rise in frailty—resulting in more strokes, of higher severity, with attenuated responses to current treatments—will require changes to current practice. Rather than considering the stroke in isolation, a holistic approach is required that includes bundles of care to manage frailty alongside rehabilitation of the stroke itself. Some of us work in clinical practice where neurologists and geriatricians jointly run stroke services, reflecting the need to have “frailty-centred” care from the outset.

Consistent with the dose-dependent effect of frailty on poor outcomes seen across acute medical presentations, increasing severity of frailty is frequently associated with deteriorating health trajectory and increasing probability of hospital admission prior to stroke.^2,79^ Explicit recognition of the overall health trajectory for individuals living with frailty may provide an opportunity to improve the personalized care of individuals approaching the end of their life. For example, a single-center study found that using frailty status in stroke survivors to trigger Palliative Care input resulted in more individuals being seen and an increase in reported competency in symptom management among healthcare staff.^80^ However, this benefit is likely to be limited by the global availability of Palliative Care practitioners, particularly outside of Europe. Given the anticipated increased burden of both frailty and stroke, increasing global access to Palliative Care services is critical.

At a global level, the World Health Organization guidance to achieve Integrated Care for Older People (ICOPE) involves a person-centered assessment and pathways in primary care, that may include multiple interventions to manage declines in intrinsic capacity, provide social care and support, support self-management, train healthcare staff, and support caregivers.^81^ This approach emphasizes timely recognition and addressing of emerging frailty in an effort to slow or arrest its development.

## Conclusions

**Figure 4.**
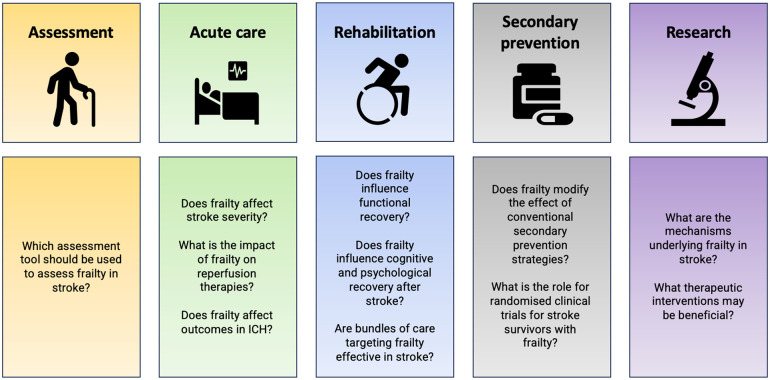
The impact of frailty at different stages of the stroke pathway.

The predicted rise in both stroke and frailty with the global aging population means that a better understanding of the impact of frailty on all stages of the stroke pathway is essential (Figure 4). The increasing incorporation of the measurement of frailty in routine and research practice will be an essential start in closing the current evidence-practice gap. With increased recognition, measurement and focused interventions in future research and clinical practice, we will generate the necessary evidence on how best to manage, and indeed treat, concurrent frailty in order to mitigate its effects and promote healthy aging within stroke care.
